# Continuous intratracheal gas suctioning combined with ventilator leak-compensation may enhance carbon dioxide removal efficiency: a proof-of-concept study using a porcine model

**DOI:** 10.1186/s40635-026-00936-y

**Published:** 2026-07-01

**Authors:** Norihiko Tsuboi, Kaoru Tsuboi, Shin Tsubokura, Taku Miyasho, Takaharu Itami, Muneyuki Takeuchi

**Affiliations:** 1 Department of Critical Care and Anesthesia, National Center for Child Health and Development, 2-10-1 Okura, Setagaya, Tokyo, 157-8535 Japan; 2 Laboratory of Animal Biological Responses, Department of Veterinary Science, Rakuno Gakuen University, 582 Bunkyodaimidorimachi, Ebetsu, 069-8501 Hokkaido Japan; 3 Laboratory of Veterinary Anesthesiology, Department of Veterinary Medicine, Rakuno Gakuen University, 582 Bunkyodaimidorimachi, Ebetsu, 069-8501 Hokkaido Japan; 4 Department of Critical Care Medicine, National Cerebral and Cardiovascular Center, 6-1 Kishibeshinmachi, Suita, 564-8565 Osaka Japan

**Keywords:** Tracheal gas insufflation, Aspiration of expiratory gas in the dead space, Mechanical ventilation, Carbon dioxide removal, Dead space, Pressure-controlled ventilation, Leak compensation

## Abstract

**Background:**

Although tracheal gas insufflation (TGI) and aspiration of expiratory gas in the dead space (ASPIDS) have been shown to enhance CO₂ removal by washing out the anatomical dead space, clinical implementation has been limited due to complications including unintended airway pressure increases and mucosal damage, as well as their complexity. We developed a novel respiratory management system combining continuous intratracheal gas suctioning with pressure control ventilation, utilizing the leak compensation function of modern ventilators. The aim of this study was to document the preliminary physiological observations of this system in a proof-of-concept porcine model.

**Methods:**

Two female pigs weighing approximately 30 kg were intubated with 7.5 mm cuffed endotracheal tubes and paralyzed under general anesthesia. Each pig was studied under four different ventilatory conditions: two with healthy lungs and two with experimentally induced lung injury (created by repeated whole-lung lavage with normal saline and injurious ventilation until PaO₂/F_I_O₂ < 300 mmHg). A 10 Fr closed suction catheter was inserted into the trachea. Using the leak compensation function of a Puritan Bennett™ 840 ventilator, heated and humidified gas was automatically supplied at the same flow rate as the continuous suctioning flow (13–14 L/min), maintaining constant circuit pressure. Pressure-controlled ventilation was applied, and arterial blood gas was analyzed before, during, and after continuous suctioning. PEEP was increased by 1 cmH₂O during suctioning to compensate for pressure loss through the endotracheal tube.

**Results:**

A total of eight experimental runs were performed (two pigs × four conditions each: two healthy, two injured lung conditions). Baseline PaCO₂ ranged from 42.2 to 71.8 mmHg. During suctioning, PaCO₂ decreased in seven conditions (reductions: 0.9 to 10.1 mmHg; 1.8% to 14.1%) and increased in one condition (3.0 mmHg; 5.9%). The largest reductions occurred in conditions with higher baseline PaCO₂. After cessation of suctioning, PaCO₂ returned toward baseline levels. No ventilator alarms or operational problems occurred during the experiments.

**Conclusions:**

In a proof-of-concept study using a porcine model with both healthy and injured lungs, continuous intratracheal gas suctioning combined with ventilator leak-compensation may enhance CO₂ removal efficiency without increasing driving pressure. This technique has potential as a simple adjunct to conventional mechanical ventilation for enhancing CO₂ removal in patients with respiratory failure, while further investigation is required for safety and clinical applicability.

**Supplementary Information:**

The online version contains supplementary material available at 10.1186/s40635-026-00936-y.

## Introduction

Mechanical ventilation is a life-saving intervention for patients with respiratory failure, but it can cause ventilator-induced lung injury (VILI) through excessive tidal volumes and pressures [1]. Lung-protective ventilation strategies aim to minimize VILI by limiting tidal volumes and plateau pressures, but this approach often results in hypercapnia [2]. Although mild hypercapnia is generally well-tolerated, severe hypercapnia can lead to adverse physiological effects, including intracranial hypertension, pulmonary hypertension, and myocardial dysfunction [3].

Tracheal gas insufflation (TGI) was developed several decades ago as an adjunctive technique to enhance CO₂ removal efficiency during mechanical ventilation [4–6]. TGI involves the continuous or intermittent insufflation of fresh gas through a catheter positioned in the trachea, which washes out CO₂-rich gas from the anatomical dead space. Multiple studies have examined the determinants of effectiveness and diverse implementation methodologies, and demonstrated that TGI can reduce PaCO₂ by 10%-30% without increasing tidal volume or airway pressure [4–9]. However, TGI has not been widely adopted in clinical practice due to several limitations, including the risk of unintended airway pressure increases, measurement errors in expiratory tidal volume, auto-positive end-expiratory pressure (PEEP) formation, incorrect triggering, lack of humidification, and potential mucosal damage from jet flow [10]. An alternative approach, aspiration of expiratory gas in the dead space (ASPIDS), was developed by Jonson and colleagues, utilizing expiratory-phase aspiration of CO₂-rich gas from the proximal airway rather than gas insufflation [11]. Studies have shown that aspiration during the latter half of expiration is efficient for CO₂ removal, as this period contains the highest CO₂ concentration gas from the anatomical dead space. While ASPIDS avoids some complications of TGI such as auto-PEEP formation and mucosal injury from jet flow, it requires breath-phase synchronization and external fresh gas supply to the ventilator circuit, making implementation complex.

Recent advances in ventilator technology, particularly the development of sophisticated leak compensation functions, may provide an opportunity to overcome the limitations of traditional TGI and ASPIDS. Modern ventilators can automatically adjust inspiratory flow to maintain circuit pressure in the presence of leaks, such as those occurring during non-invasive ventilation or when using uncuffed endotracheal tubes [12].

We hypothesized that a novel approach—continuous suctioning of intratracheal gas combined with simultaneous automated fresh gas insufflation using the ventilator’s leak compensation function of modern ICU ventilators—could provide a simpler, safer alternative to traditional TGI and ASPIDS. We named this technique “Tracheal Humidified Rapid Insufflation with Continuous Suctioning” (THRICS).

The aim of this study was to document preliminary physiological observations of continuous intratracheal gas suctioning combined with ventilator leak compensation function in a porcine model, as a first step toward establishing the conceptual basis for this technique.

## Methods

### Ethical approval

This study was an experimental investigation using a porcine model. The experiment was conducted at Rakuno Gakuen University, Hokkaido, Japan, which is equipped with animal experiment facilities. Rakuno Gakuen University has established institutional regulations for animal experiments based on the Guide for the Care and Use of Laboratory Animals [13]. This experiment was carried out in accordance with these provisions and approved by the Ethics Committee of Rakuno Gakuen University (approval number: VH25B5) and ARRIVE guidelines 2.0 [14].

### Experimental respiratory management system

**Fig. 1:**
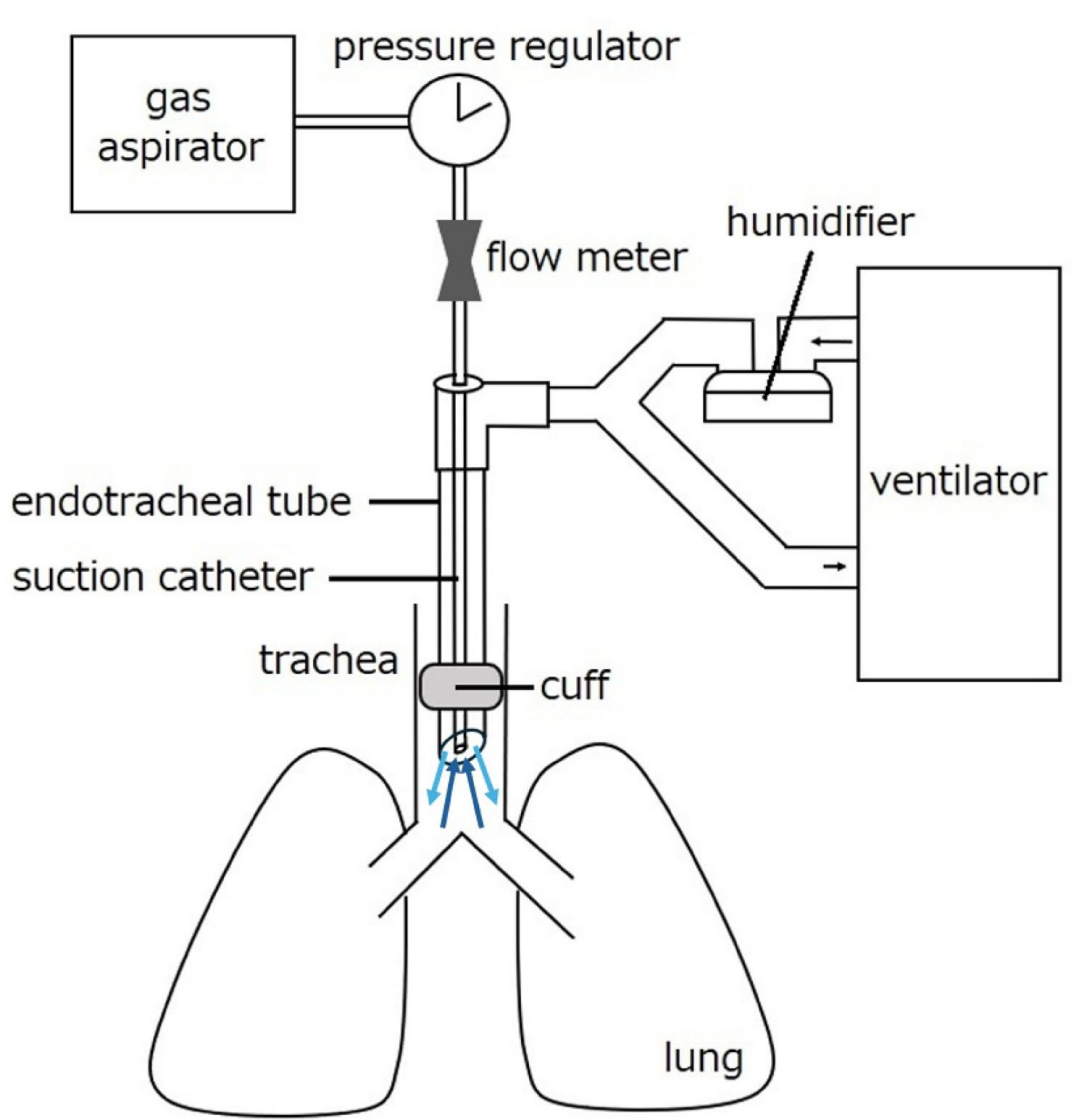
Conceptual diagram of the THRICS (Tracheal Humidified Rapid Insufflation with Continuous Suctioning) respiratory management system. A closed suction catheter is inserted through the endotracheal tube into the trachea and connected to a suction source via a flow meter. The ventilator’s leak compensation function automatically delivers heated and humidified fresh gas at a rate equal to the continuous suctioning flow rate, maintaining constant circuit pressure. This results in continuous replacement of CO₂-rich gas in the anatomical dead space

The conceptual design of the respiratory management system used in this experiment is shown in Fig. 1. A closed-type small-diameter suction catheter (TRACH CARE™, 10 Fr, Avanos Medical Japan, Inc., Yokohama, Japan) was inserted through the endotracheal tube into the trachea. The suction catheter was connected to a wall suction source via a flow meter, allowing precise control of the continuous suctioning flow rate. FlowAnalyser PF-300 and the dedicated software FlowLab (IMT Japan, Inc., Nagoya, Japan) was used for flow measurement.

The endotracheal tube was connected to a Puritan Bennett™ 840 ventilator (Medtronic Japan, Inc., Tokyo, Japan) equipped with a heated humidifier on the inspiratory limb. The ventilator’s leak compensation function was activated, which automatically adjusts inspiratory flow to maintain the set circuit pressure even in the presence of continuous gas leakage. When continuous suctioning was initiated through the intratracheal catheter, the leak compensation function caused the ventilator to automatically increase fresh gas delivery at a rate equal to the suctioning flow rate, thereby maintaining constant circuit pressure. This resulted in continuous turnover of gas in the trachea with heated, humidified fresh gas.

### Preliminary experiment

Because continuous gas suctioning through the endotracheal tube creates a pressure gradient, we conducted preliminary experiments to determine the appropriate PEEP compensation needed to maintain similar intratracheal pressures distal to the endotracheal tube tip and expiratory tidal volumes during THRICS operation. Detailed descriptions, conditions and results of preliminary experiments are shown in the supplementary materials (Descriptions of preliminary experiments, Leak compensation algorithm, Table S1, Table S2, and Figure S1).

Based on these preliminary findings, we adopted a protocol of increasing PEEP by 1 cmH₂O and maintaining continuous suctioning at 13–14 L/min for all experimental conditions to ensure that intratracheal pressure distal to the endotracheal tube tip and actual expiratory tidal volume delivery remained comparable before and during THRICS operation.

### Animal preparation and anesthetic management

Two female pigs (pig A and pig B) weighing approximately 30 kg at three months of age were used for this study. Each pig underwent four experimental conditions (two with healthy lungs and two with injured lungs), resulting in a total of eight experimental runs. The anesthetic protocol was standardized for all experiments:

1. **Premedication**: Midazolam (0.2 mg/kg), butorphanol (0.2 mg/kg), and medetomidine (40 µg/kg) were administered intramuscularly.
2. **Induction**: After securing peripheral venous access via the ear vein, propofol (3 mg/kg) was administered intravenously for induction of anesthesia.
3. **Intubation**: The pig was intubated with a 7.5 mm inner diameter cuffed endotracheal tube using a laryngoscope.
4. **Monitoring**: The pig was positioned supine, and an arterial catheter was inserted into the femoral artery for continuous blood pressure monitoring and arterial blood gas sampling.
5. **Maintenance anesthesia**: General anesthesia was maintained with continuous intravenous infusions of propofol (12 mg/kg/h), medetomidine (5 µg/kg/h), and rocuronium (1.5-2 mg/kg/h), with additional boluses administered as needed to maintain adequate anesthesia and muscle relaxation.

### Lung injury model

For each pig, experiments were first conducted with healthy lungs under two different ventilatory conditions. Subsequently, lung injury was induced using repeated whole-lung lavage with warmed normal saline (500 mL per lavage cycle), a well-established model of acute lung injury [15, 16]. After each lavage cycle, the pigs were ventilated for 10 min using injurious ventilator settings in pressure control–assist control (PC-AC) mode (respiratory rate:10/min; inspiratory pressure: 30 cmH₂O above PEEP; PEEP: 0 cmH₂O; inspiration time: 1.0 s; F_I_O₂: 1.0) to promote atelectasis and inflammatory injury. This lavage-ventilation sequence was repeated 2–3 times until the PaO₂/F_I_O₂ ratio decreased to less than 300 mmHg, confirming the establishment of lung injury. After achieving the target degree of lung injury, experiments were conducted under two different ventilatory conditions with the injured lungs.

### Experimental protocol

After anesthetic induction and instrumentation, the following protocol was implemented:

1. **Baseline ventilation:** PC-AC mode was initiated. Ventilator settings were adjusted to establish various baseline conditions representing different degrees of alveolar ventilation efficiency.
  Healthy lung conditions:
  Condition 1
    Respiratory rate: 20/min; inspiratory pressure: 10 cmH₂O above PEEP; PEEP: 5 cmH₂O; inspiration time: 1.5 sec; F_I_O₂: 0.4
  Condition 2
    Respiratory rate: 15/min; inspiratory pressure: 14 cmH₂O above PEEP; PEEP: 5 cmH₂O; inspiration time: 2.0 sec; F_I_O₂: 0.4
  Injured lung conditions:
  Condition 3
    Respiratory rate: 20/min; inspiratory pressure: 15 cmH₂O above PEEP; PEEP: 10 cmH₂O; inspiration time: 1.5 sec; F_I_O₂: 0.6
  Condition 4
    Respiratory rate: 30/min; inspiratory pressure: 10 cmH₂O above PEEP; PEEP: 10 cmH₂O; inspiration time: 1.0 sec; F_I_O₂: 0.6

This study employed a within-subject repeated-measures design. Each animal underwent all four experimental conditions sequentially in the above order sequentially. Additional operational parameters were configured as follows: trigger sensitivity (Psens) was set to 20 cmH₂O (the least sensitive setting available) to prevent auto-triggering caused by continuous suction-induced flow within the ventilator circuit; rise time was set to 50%; and the leak compensation threshold (Dsens) was set to the maximum value of 65 L/min to ensure full compensation of the suction-induced leak throughout all experimental conditions. These settings were kept constant across all experimental runs.

2. **Catheter insertion**: During continuous suctioning, a 10 Fr closed suction catheter (TRACH CARE™, Avanos Medical Japan, Inc.) was inserted into the endotracheal tube, positioning its tip precisely at the end of the endotracheal tube. This position was maintained consistently across all experimental runs.
3. **Experimental sequence**: For each baseline ventilatory condition, the following three-phase sequence was performed:
  Phase 1 (Pre-suctioning)
    Baseline ventilation without the suction catheter in place. After at least 8 minutes of stabilization, arterial blood was sampled for gas analysis.
  Phase 2 (During-suctioning)
    The suction catheter was inserted, PEEP was increased by 1 cmH₂O, and continuous suctioning was initiated at 13-14 L/min. The leak compensation function automatically increased fresh gas delivery to match the suctioning rate. Suction was applied using a standard wall suction system with a pressure-regulated suction regulator set to below 150 mmHg, with fine manual adjustment required to achieve the target flow rate. As suction was pressure-controlled, the instantaneous suction flow rate varied throughout the respiratory cycle, being higher during inspiration and lower during expiration due to respiratory cycle-dependent changes in the pressure gradient between the airway and the suction source. Therefore, the suction flow rate was titrated based on the mean flow rate averaged over 2-3 minutes, as measured by a flow meter incorporated into the suction circuit. After at least 8 minutes of stabilization, arterial blood was sampled for gas analysis.
  Phase 3 (Post-suctioning)
    Continuous suctioning was stopped, PEEP was returned to the baseline level, and the suction catheter was withdrawn from the endotracheal tube. After at least 8 minutes of stabilization, arterial blood was sampled for gas analysis.

4. **Data collection**: Arterial blood gas samples were analyzed immediately using a point-of-care blood gas analyzer. Ventilator parameters including expiratory tidal volume, respiratory rate, peak inspiratory pressure, PEEP, mean airway pressure, and leak measurements were recorded. Hemodynamic parameters including heart rate and arterial pressure, respiratory parameters including SpO_2_ and E_T_CO_2_, and body temperature were also monitored.

### Statistical analysis

Given the limited sample size (*n* = 2 animals with multiple measurements per animal), formal statistical hypothesis testing was not performed following biostatistician consultation. This study reports descriptive statistics only. Data are presented as individual values for each experimental condition, with ranges and observed patterns described. No inferential statistics or *P* values are reported. The primary outcome was the change in PaCO₂ during continuous intratracheal suctioning compared to baseline. Statistical analyses were performed with EZR (Saitama Medical Center, Jichi Medical University, Saitama, Japan), which is a graphical user interface for R (The R Foundation for Statistical Computing, Vienna, Austria).

## Results

### Experimental system performance

The THRICS respiratory management system operated without technical problems throughout all eight experimental runs. No ventilator alarms were triggered during the experiments. The leak compensation function of the ventilator successfully maintained constant circuit pressure while automatically matching fresh gas delivery to the continuous suctioning flow rate of 13–14 L/min. Visual inspection of the ventilator display confirmed that the system detected the continuous leak and compensated appropriately. Throughout the animal experiments, we also continuously monitored the ventilator flow-time waveform display and confirmed that end-inspiratory flow reached zero in every breath.

### Changes in PaCO_2_

Table 1 shows the absolute PaCO₂ values for all eight experimental runs (two pigs × four conditions each) during the three phases: pre-suctioning (baseline), during continuous suctioning, and post-suctioning. The baseline PaCO₂ values varied across the eight runs (range: 42.2–71.8 mmHg), reflecting the intentionally different baseline ventilatory conditions, including both healthy lung and injured lung states. During continuous endotracheal suctioning, PaCO₂ decreased compared to baseline in seven of the eight experimental runs. The absolute reductions ranged from 0.9 to 10.1 mmHg. One experimental run (Condition 1A) showed an increase of 3.0 mmHg during suctioning. The magnitude of PaCO₂ reduction varied across conditions, with larger reductions generally observed in conditions with higher baseline PaCO₂ levels. After cessation of suctioning, PaCO₂ returned toward baseline levels in all eight experimental runs. The post-suctioning PaCO₂ values ranged from 38.2 to 70.0 mmHg, compared to baseline values of 42.2 to 71.8 mmHg.

**Table 1:** Absolute PaCO_2_ values (mmHg)

Experimental run	Pre-suctioning	During-suctioning	Post-suctioning
Condition 1A	50.9	53.9	53.5
Condition 1B	49.7	48.8	49.8
Condition 2A	47.1	42.2	43.3
Condition 2B	42.2	39.5	38.2
Condition 3A	61.1	58.9	62.2
Condition 3B	56.9	50.7	52.7
Condition 4A	71.8	61.7	70.0
Condition 4B	62.4	57.8	64.2

The A and B following the condition number represent two different pigs, each tested under four different ventilatory conditions (Conditions 1–4: two with healthy lungs and two with injured lungs). During suctioning, PaCO₂ decreased in seven conditions (reductions: 0.9 to 10.1 mmHg) and increased in one condition (3.0 mmHg)

Table 2 shows the relative changes in PaCO₂ expressed as percentages of baseline values. During continuous suctioning, relative PaCO₂ reductions ranged from 1.8% to 14.1% in the seven runs that showed decreases, while one run showed a 5.9% increase. After cessation of suctioning, PaCO₂ values returned to 90.5% to 105.1% of baseline across all runs.

**Table 2:** Relative PaCO_2_ changes (% of baseline)

Experimental run	Pre-suctioning	During-suctioning	Post-suctioning
Condition 1A	100	105.9	105.1
Condition 1B	100	98.2	100.2
Condition 2A	100	89.6	91.9
Condition 2B	100	93.6	90.5
Condition 3A	100	96.4	101.8
Condition 3B	100	89.1	92.6
Condition 4A	100	85.9	97.5
Condition 4B	100	92.6	102.9

The A and B following the condition number represent two different pigs, each tested under four different ventilatory conditions (Conditions 1–4: two with healthy lungs and two with injured lungs). During suctioning, PaCO₂ decreased in seven conditions (reductions: 1.8% to 14.1%) and increased in one condition (5.9%)

**Fig. 2:**
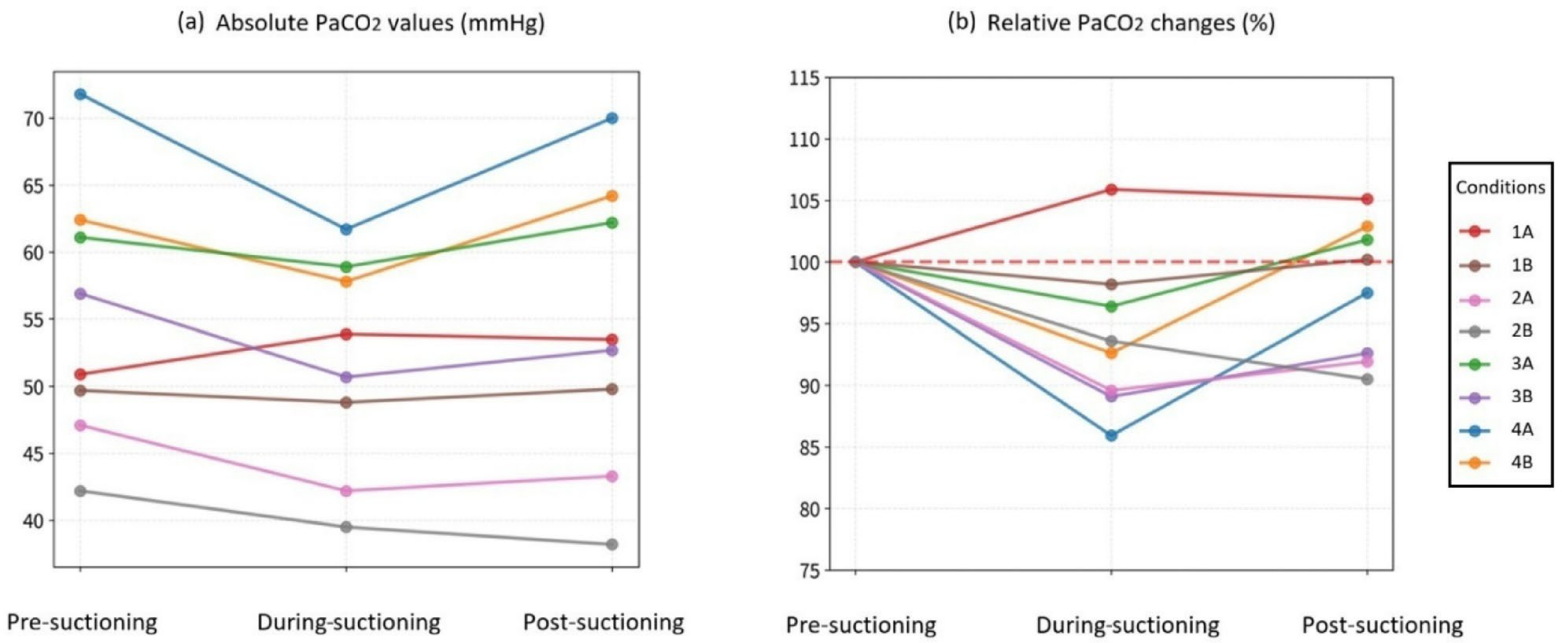
Changes in PaCO₂ across experimental phases. (**a**) Absolute PaCO₂ values (mmHg) for all eight experimental runs during pre-suctioning (baseline), during continuous intratracheal suctioning, and post-suctioning phases. (**b**) Relative PaCO₂ changes expressed as percentage of baseline values

Figure 2 illustrates the changes in PaCO₂ across the three phases for all eight experimental runs, showing both the absolute values (Fig. 2a) and the percentage changes relative to baseline (Fig. 2b).

### Pattern by animal

In Pig A, PaCO₂ decreased during suctioning in three of four conditions (reductions: 2.2 to 10.1 mmHg) and increased in one condition (3.0 mmHg). In Pig B, PaCO₂ decreased during suctioning in all four conditions (reductions: 0.9 to 6.2 mmHg).

### Pattern by lung state

In healthy lung conditions (Conditions 1A, 1B, 2A, 2B), PaCO₂ decreased in three of four runs (range: 0.9 to 4.9 mmHg decrease; one 3.0 mmHg increase). In injured lung conditions (Conditions 3A, 3B, 4A, 4B), PaCO₂ decreased in all four runs (range: 2.2 to 10.1 mmHg).

### Lung injury confirmation

For the lung injury experiments, successful establishment of lung injury was confirmed by PaO₂/F_I_O₂ ratio < 300 mmHg after the lavage-ventilation procedures. In the conditions of injured lungs, adequate oxygenation was maintained by using higher PEEP (10 cmH₂O) and higher F_I_O₂ (0.6) compared to the condition of healthy lungs (PEEP 5 cmH₂O, F_I_O₂ 0.4).

### Other blood gas parameters

Partial pressure of arterial oxygen (PaO₂) was maintained at adequate levels throughout all experiments using appropriate F_I_O₂ settings (0.4 for healthy lungs, 0.6 for injured lungs). Arterial pH showed minor changes corresponding to the PaCO₂ alterations, but all values remained within the physiological range. No episodes of significant hypoxemia or severe acidosis/alkalosis occurred during the experiments. During the eight test runs, PaO₂ ranged from 163 mmHg to 234 mmHg, and pH ranged from 7.283 to 7.542 (see Supplementary Tables S3 and S4).

### Hemodynamic stability

Heart rate and mean arterial blood pressure remained stable throughout all experimental phases, with no clinically significant changes associated with the initiation or cessation of continuous suctioning. No adverse hemodynamic events occurred during any of the experiments.

## Discussion

This study provides preliminary physiological observations suggesting that continuous intratracheal gas suctioning combined with ventilator leak compensation function under PC-AC ventilation may affect CO₂ removal in a porcine model. In seven of eight experimental runs across two pigs under varying ventilatory conditions (healthy and injured lungs), continuous suctioning at 13–14 L/min reduced PaCO₂ without increasing driving pressure.

### Mechanism of enhanced CO_2_ removal

The mechanism likely involves continuous replacement of CO₂-rich gas in the anatomical dead space with fresh gas. During normal tidal ventilation, a portion of each breath ventilates the anatomical dead space (endotracheal tube, trachea, and conducting airways) rather than participating in alveolar gas exchange. In our THRICS system, continuous suctioning at 13–14 L/min creates steady outward flow through the suction tube, while the ventilator’s leak compensation function simultaneously delivers fresh humidified gas at an equal rate, effectively washing out CO₂-rich gas from the anatomical dead space, including the dead space of the endotracheal tube. However, flushing out the physiological dead space of the alveolar component is considered difficult by THRICS. Therefore, in conditions such as acute respiratory distress syndrome (ARDS) or chronic obstructive pulmonary disease (COPD) where the physiological dead space in the alveoli increases, the CO_2_ removal effect may be restricted.

### Comparison with traditional TGI and ASPIDS

Traditional TGI involves the insufflation of fresh gas through a catheter into the trachea, typically at flow rates of 2–10 L/min [4–9]. Studies have reported that TGI can reduce PaCO₂ by 10%-30% depending on the flow rate and catheter position [5, 6]. In comparison, our study demonstrated a more modest 1.8% to 14.1% reduction in PaCO₂ with THRICS. This difference in efficacy may be explained by differences in gas flow dynamics: TGI delivers fresh gas through a catheter positioned near the carina, creating a forward jet flow that actively propels fresh gas distally into the bronchial tree and potentially washes out CO₂-rich gas from deeper anatomical dead space. In contrast, THRICS creates a negative pressure gradient at the proximal trachea by continuous suctioning, which primarily affects gas in the trachea and proximal airways with limited effect on more distal bronchial dead space. On the other hand, there are reports indicating that ASPIDS, which shares the same mechanism of intratracheal suctioning as THRICS, demonstrated comparable CO₂ removal capacity to phasic TGI [17]. Given the small sample size in this study, the extent of its effectiveness requires further verification.

Although its CO_2_ removal effect may be modest, THRICS offers several practical advantages over both TGI and ASPIDS. First, THRICS requires no external gas supply, or breath-phase synchronization equipment, as fresh gas is provided automatically through the ventilator’s leak-compensation mechanism via the heated humidifier circuit. Second, unlike TGI, the negative-pressure suction of THRICS avoids unintended increases in airway pressure, jet-flow mucosal injury, and interference with ventilator flow sensors. Third, unlike ASPIDS, THRICS does not require mandatory synchronization with the respiratory cycle, substantially simplifying implementation and theoretically enabling its application even in the presence of spontaneous breathing. These characteristics suggest that THRICS may be more readily implementable at the bedside in routine clinical practice.

### Airway resistance and inspiratory time considerations

An important practical consideration when implementing THRICS is the potential increase in airway resistance at the endotracheal tube level. Two mechanisms contribute to this increase: first, the physical presence of the suction catheter within the endotracheal tube reduces the effective cross-sectional area available for gas flow; second, the leak-compensation mechanism generates a continuous inward additional flow within the endotracheal tube.

This increased resistance has two clinically relevant consequences. First, it prolongs the respiratory time constant, meaning that if inspiratory time is insufficient, circuit pressure may not be fully transmitted to the lungs before inspiration ends, potentially resulting in reduced effective tidal volume delivery despite unchanged ventilator pressure settings. In this study, we carefully monitored this issue by continuously observing the flow-time waveform and confirmed that all breaths reached zero end-inspiratory flow under all experimental conditions, indicating adequate pressure equilibration throughout. Second, the continuous inward flow and the increased resistance cause a pressure loss at the endotracheal tube level, resulting in a discrepancy between the ventilator circuit pressure and the actual distal airway pressure. Although the leak-compensation function maintains circuit pressure at the set values, this endotracheal tube level pressure loss means that pressure at the distal airway is reduced relative to the ventilator settings. In this study, we addressed this issue by manually increasing PEEP by 1 cmH₂O, which our preliminary bench experiments confirmed precisely compensated for this pressure loss, as verified by distal airway pressure measurement and expiratory tidal volume measurement by external flow analyzer (Supplementary Tables S1-S2).

When considering future clinical application of THRICS, clinicians should be aware of both consequences of increased endotracheal tube resistance: the need for adequate inspiratory time to ensure complete tidal volume delivery, and the need for appropriate PEEP adjustment to compensate for endotracheal tube level pressure loss. Patients with high airway resistance (e.g., those with COPD or bronchospasm) or those requiring precise PEEP adjustment (e.g., severe ARDS) require particularly careful monitoring of ventilator waveforms during THRICS implementation, with attention to decreases in distal airway pressure relative to circuit pressure.

### Clinical implications and potential applications

The findings of this proof-of-concept study have limited direct clinical applicability at this stage. However, the observed pattern of greater CO₂ reduction at higher baseline PaCO₂ levels suggests that THRICS may be relevant in hypercapnic patients in whom conventional lung-protective ventilation strategies result in permissive hypercapnia, such as those with ARDS or severe obstructive lung disease. In such patients, even a modest reduction in PaCO₂ without an increase in driving pressure could potentially reduce the need for escalation to extracorporeal CO₂ removal. That said, given the very small sample size, absence of formal safety data, and short observation period of the current study, clinical application cannot be recommended until larger studies with systematic safety assessments have been conducted. It must be emphasized that all interpretations in this discussion are necessarily speculative, given that the dataset comprises observations from only two individual animals.

### Limitations

This study has the following important limitations that should be acknowledged:

1. **Very small sample size with limited generalizability**: This study used only two animals, each of which was tested under four different ventilatory conditions. Differences in PaCO₂ response to THRICS were also observed between individuals (pig A and pig B). We determined that formal statistical testing was not appropriate for this dataset, and results are therefore presented as descriptive data only. The findings should be considered preliminary proof-of-concept data only, and confirmation in larger, adequately powered studies with independent subjects is required before any conclusions can be drawn. Consequently, the data presented in this manuscript are more appropriately characterized as preliminary physiological observations from individual experimental runs than as reproducible experimental results.
2. **Short observation period and incomplete tissue CO₂ equilibration**: Each experimental phase lasted at least 8 minutes, which was insufficient for complete tissue CO₂ washout to steady state. Tissue CO₂ stores buffer acute changes in ventilatory efficiency, and complete equilibration requires 20-120 minutes [18]. However, extending observation periods in paralyzed animals posed practical challenges including progressive changes in lung mechanics (atelectasis development or recruitment), potential hemodynamic instability during prolonged anesthesia, and difficulty distinguishing THRICS effects from time-dependent physiological changes. Future studies should include longer observation periods with careful monitoring to assess THRICS efficacy at truly steady state.
3. **Species differences**: Porcine respiratory anatomy and physiology differ from humans in several respects, including airway dimensions, dead space-to-tidal volume ratio, respiratory mechanics, and ventilatory control responses. These differences may affect THRICS efficacy and safety. Clinical studies are needed to confirm efficacy and safety in human patients before any clinical application can be considered.
4. **Lung injury model limitations:** The lavage-induced lung injury model produces primarily atelectasis and surfactant depletion, which may not fully replicate the inflammatory changes, fibroproliferative responses, ventilation-perfusion mismatch patterns, and increased alveolar dead space characteristics of clinical ARDS. The acute injury induced in our study does not capture chronic changes in clinical disease states. As discussed, THRICS efficacy may be reduced in conditions with extensive alveolar dead space, which our injury model may not adequately reproduce. Future studies should test THRICS in animal models with greater V/Q heterogeneity and alveolar dead space.
5. **Single ventilator mode tested**: This study examined PC-AC mode only, with animals fully paralyzed. THRICS efficacy and safety with other ventilation modes (volume-controlled, pressure support, high-frequency oscillatory ventilation, etc.) and during spontaneous breathing efforts remain unknown. The potential compatibility of THRICS with spontaneous breathing modes requires dedicated investigation.
6. **Suboptimal catheter positioning and suction flow rate**: The suction catheter tip was positioned at the distal end of the endotracheal tube, placing the suction inlet in close proximity to the fresh gas outlet from the leak compensation system. This positioning, combined with continuous suction at 13-14 L/min (particularly during late expiration when expiratory flow decreases), may result in suctioning fresh compensatory gas rather than CO₂-rich expiratory gas, reducing efficiency and increasing gas consumption (approximately 800 L/hour). The optimal spatial relationship between catheter tip and endotracheal tube tip, as well as the optimal suction flow rate, are currently unknown.
7. **No histological safety assessment**: In this study, microscopic mucosal changes and effects on mucociliary function were not assessed. Future studies should include histological examination of tracheal and bronchial mucosa, assessment of mucociliary clearance, and measurement of inflammatory markers to comprehensively evaluate airway safety during prolonged THRICS operation.
8. **Absence of lung volume and regional ventilation assessment**: This study did not include direct assessment of end-expiratory lung volume, regional ventilation distribution, or lung volume changes during THRICS. Electrical impedance tomography (EIT) would be a particularly valuable tool, enabling non-invasive, real-time assessment of regional ventilation distribution and end-expiratory lung volume changes during THRICS.

### Future research directions

Key questions for future research include:

1. Optimization studies: computational fluid dynamics modeling to guide optimization of catheter positioning, followed by experimental evaluation of the dose-response relationships for suctioning flow rate, the effects of catheter position relative to endotracheal tube tip, and humidification adequacy assessment through direct distal airway measurements.
2. Long-term safety: effects on airway mucosa, mucociliary clearance, and ventilator-associated pneumonia risk with histological examination.
3. Diseased lung models: efficacy in animal models of ARDS or COPD where increased dead space may enhance dead space washout benefits.
4. Alternative ventilation modes and spontaneous breathing compatibility: crucial investigations for clinical applicability, particularly pressure support ventilation and other spontaneous modes where THRICS may offer advantages over conventional TGI and ASPIDS [19].
5. Advanced measurement techniques: EIT studies for accurate tidal volume quantification and regional ventilation assessment.
6. Clinical trials: carefully designed trials in selected populations (e.g., ARDS requiring lung-protective ventilation) to assess clinical outcomes.
7. Device development: development of integrated systems (double-lumen tubes, flow-controlled suction system, ventilators with automatic THRICS control and accurate volume measurement).

## Conclusions

In this proof-of-concept study using porcine model, continuous intratracheal gas suctioning combined with ventilator leak-compensation under PC-AC ventilation reduced PaCO₂ in seven of eight experimental runs without increasing driving pressure. This novel technique utilizes the leak-compensation function of modern ventilators, potentially addressing some of the practical limitations of traditional TGI and ASPIDS. However, given the very small sample size and the absence of formal safety assessments, these findings should be interpreted as preliminary only. Future research should focus on optimizing technical parameters, conducting systematic safety evaluations including airway mucosal assessment, and evaluating efficacy in larger animal cohorts before clinical translation.

## Supplementary Information

Below is the link to the electronic supplementary material.

Supplementary Material 1

## Data Availability

The datasets used and/or analyzed during the current study are available from the corresponding author on reasonable request.

## Abbreviations

VILI: Ventilator-induced lung injury
TGI: Tracheal gas insufflation
ASPIDS: Aspiration of expiratory gas in the dead space
PEEP: Positive end-expiratory pressure
THRICS: Tracheal humidified rapid insufflation with continuous suctioning
PC-AC: Pressure control–assist control
ARDS: Acute respiratory distress syndrome
COPD: Chronic obstructive pulmonary disease
EIT: Electrical impedance tomography
