# Supplementary material for: Continuous intratracheal gas suctioning combined with ventilator leak-compensation may enhance carbon dioxide removal efficiency: a proof-of-concept study using a porcine model
Source: Intensive Care Med Exp. 2026 Jul 1;14:83. doi: 10.1186/s40635-026-00936-y (PMC13323452; doi:10.1186/s40635-026-00936-y)
Supplement: Supplementary file 1 — Supplementary Material 1 [file 40635_2026_936_MOESM1_ESM.docx]

**Supplementary files**

Descriptions of preliminary experiments

An artificial lung capable of altering compliance and resistance (SmartLung™, IMT Japan, Inc., Nagoya, Japan) was attached to the distal end of a 7.5 mm cuffed endotracheal tube. Pressure control–assist control (PC-AC) ventilation was applied, and the pressure distal to the endotracheal tube tip was measured with and without continuous suctioning at various flow rates and PEEP settings. During continuous suctioning, a 10 Fr suction catheter (TRACH CARE™, Avanos Medical Japan, Inc., Yokohama, Japan) was inserted into the endotracheal tube, positioning its tip precisely at the end of the endotracheal tube. This position was maintained consistently across all preliminary experimental runs. The schematic diagram for the preliminary experiments is shown in Figure S1.

The results demonstrated that continuous suctioning at 13-14 L/min caused a pressure loss of approximately 1 cmH₂O across the endotracheal tube. By increasing PEEP by 1 cmH₂O during continuous suctioning, we were able to maintain the mean intratracheal pressure distal to the endotracheal tube tip at levels similar to baseline without suctioning. Furthermore, when inspiratory time was sufficient and inspiratory driving pressure remained constant, the actual expiratory tidal volume delivered to the artificial lung remained essentially unchanged between conditions with and without continuous suctioning (with appropriate PEEP compensation). Continuous suction rate and actual expiratory tidal volume were also measured by FlowAnalyser PF-300 with dedicated software FlowLab (IMT Japan, Inc., Nagoya, Japan). Additionally, it was found that the expiratory tidal volume measured by the internal sensor of the ventilator was consistently lower than the actual delivered volume during continuous suction, even when the leak compensation function was used. Suction was applied using a standard wall suction system with a pressure-regulated suction regulator set to below 150 mmHg, with fine manual adjustment required to achieve the target flow rate. As suction was pressure-controlled, the instantaneous suction flow rate varied throughout the respiratory cycle, being higher during inspiration and lower during expiration due to respiratory cycle-dependent changes in the pressure gradient between the airway and the suction source. Therefore, the suction flow rate was titrated based on the mean flow rate averaged over 2-3 minutes, as measured by a flow meter incorporated into the suction circuit. Detailed conditions and results of the preliminary experiments are shown in the supplementary materials (Table S1, Table S2, and Figure S2).

Leak compensation algorithm

The PB840 leak compensation algorithm operates during expiratory phase. During expiration, it delivers additional fresh gas to maintain the set PEEP level in the ventilator circuit. During inspiration in PC-AC mode, the ventilator delivers flow until circuit pressure reaches the set inspiratory pressure regardless of leak presence. As a result, ventilator circuit pressure is maintained at the set values during both inspiratory and expiratory phases through the combined action of pressure-controlled inspiration and active leak compensation during expiration.

Table S1. Preliminary experimental conditions

| Preliminary experimental run | Ventilator settings | | | | | | Artificial lung settings | | THRICS |
| --- | --- | --- | --- | --- | --- | --- | --- | --- | --- |
|  | Mode | Respiratory rate (/min) | Inspiratory pressure above PEEP (cmH_2_O) | PEEP (cmH_2_O) | Inspiration time (s) | F_I_O_2_ | Compliance (mL/mbar) | Resistance (mbar/L/s) |  |
| Condition 1-1a | PC-AC | 20 | 10 | 5 | 1.5 | 0.4 | 30 | 5 | **−** |
| Condition 1-1b | PC-AC | 20 | 10 | 6 | 1.5 | 0.4 | 30 | 5 | **+** |
| Condition 1-2a | PC-AC | 20 | 10 | 5 | 1.5 | 0.4 | 30 | 5 | **−** |
| Condition 1-2b | PC-AC | 20 | 10 | 6 | 1.5 | 0.4 | 30 | 5 | **+** |
| Condition 2-1a | PC-AC | 15 | 14 | 5 | 2.0 | 0.4 | 30 | 5 | **−** |
| Condition 2-1b | PC-AC | 15 | 14 | 6 | 2.0 | 0.4 | 30 | 5 | **+** |
| Condition 2-2a | PC-AC | 15 | 14 | 5 | 2.0 | 0.4 | 30 | 5 | **−** |
| Condition 2-2b | PC-AC | 15 | 14 | 6 | 2.0 | 0.4 | 30 | 5 | **+** |
| Condition 3-1a | PC-AC | 20 | 15 | 10 | 1.5 | 0.6 | 20 | 5 | **−** |
| Condition 3-1b | PC-AC | 20 | 15 | 11 | 1.5 | 0.6 | 20 | 5 | **+** |
| Condition 3-2a | PC-AC | 20 | 15 | 10 | 1.5 | 0.6 | 20 | 5 | **−** |
| Condition 3-2b | PC-AC | 20 | 15 | 11 | 1.5 | 0.6 | 20 | 5 | **+** |
| Condition 4-1a | PC-AC | 30 | 10 | 10 | 1.0 | 0.6 | 20 | 5 | **−** |
| Condition 4-1b | PC-AC | 30 | 10 | 11 | 1.0 | 0.6 | 20 | 5 | **+** |
| Condition 4-2a | PC-AC | 30 | 10 | 10 | 1.0 | 0.6 | 20 | 5 | **−** |
| Condition 4-2b | PC-AC | 30 | 10 | 11 | 1.0 | 0.6 | 20 | 5 | **+** |

PC-AC, pressure control–assist control; PEEP, positive end-expiratory pressure; THRICS, tracheal humidified rapid insufflation with continuous suctioning.

Subscript “a” indicates the condition without THRICS, whereas subscript “b” indicates the condition with THRICS. During continuous intratracheal suctioning, increase the PEEP setting by 1 cmH₂O, but leave all other settings unchanged.

Table S2. Preliminary experimental results

| Preliminary experimental run | Measurements on the ventilator | | | | Measurements on the flow analyzer | | | | | THRICS |
| --- | --- | --- | --- | --- | --- | --- | --- | --- | --- | --- |
|  | Expiratory tidal volume (mL) | %LEAK (%) | LEAK (L/min) | V LEAK (mL) | Pressure distal to endotracheal tube tip (cmH_2_O) | | | Expiratory tidal volume (mL) | Mean suction flow rate (L/min) |  |
|  |  |  |  |  | Maximum | Minimum | Mean |  |  |  |
| Condition 1-1a | 164 | 0 | 0 | 0 | 15.24 | 6.56 | 10.68 | 137 | 0 | **−** |
| Condition 1-1b | 123 | 75 | 8.0 | 314 | 15.38 | 6.73 | 10.61 | 127 | 13.00 | **+** |
| Condition 1-2a | 145 | 11 | 0.4 | 16 | 15.09 | 6.56 | 10.67 | 138 | 0 | **−** |
| Condition 1-2b | 124 | 66 | 5.5 | 218 | 15.48 | 6.86 | 10.71 | 144 | 13.85 | **+** |
| Condition 2-1a | 319 | 0 | 0 | 0 | 19.31 | 6.58 | 11.00 | 265 | 0 | **−** |
| Condition 2-1b | 262 | 69 | 8.0 | 470 | 19.11 | 6.44 | 11.89 | 267 | 13.30 | **+** |
| Condition 2-2a | 330 | 11 | 0.5 | 30 | 19.14 | 6.41 | 12.49 | 300 | 0 | **−** |
| Condition 2-2b | 269 | 57 | 5.3 | 313 | 19.28 | 6.77 | 12.66 | 297 | 14.04 | **+** |
| Condition 3-1a | 246 | 0 | 0 | 0 | 25.51 | 11.77 | 16.28 | 210 | 0 | **−** |
| Condition 3-1b | 218 | 62 | 8.7 | 323 | 25.47 | 11.89 | 18.06 | 216 | 13.29 | **+** |
| Condition 3-2a | 231 | 11 | 0.5 | 24 | 25.33 | 11.44 | 18.02 | 220 | 0 | **−** |
| Condition 3-2b | 210 | 53 | 5.9 | 222 | 25.59 | 11.94 | 18.07 | 219 | 14.00 | **+** |
| Condition 4-1a | 135 | 2 | 0.1 | 7 | 20.39 | 11.73 | 15.94 | 108 | 0 | **−** |
| Condition 4-1b | 105 | 68 | 9.3 | 210 | 20.29 | 11.91 | 15.40 | 103 | 13.63 | **+** |
| Condition 4-2a | 122 | 11 | 0.6 | 14 | 20.25 | 11.44 | 15.60 | 111 | 0 | **−** |
| Condition 4-2b | 105 | 60 | 6.3 | 143 | 20.52 | 11.94 | 15.83 | 111 | 14.40 | **+** |

THRICS, tracheal humidified rapid insufflation with continuous suctioning.

The subscript “a” indicates the condition without THRICS, whereas the subscript “b” indicates the condition with THRICS. Measurements on the ventilator show a decrease in tidal volume with THRICS compared to without THRICS. However, the actual tidal volume measured on the flow analyzer shows little change. Furthermore, the maximum, minimum, and mean values of the pressure distal to the endotracheal tube tip measured on the flow analyzer are nearly identical with and without THRICS.

Table S3. Absolute PaO_2_ values (mmHg)

| Experimental run | Pre-suctioning | During-suctioning | Post-suctioning |
| --- | --- | --- | --- |
| Condition 1A | 173 | 174 | 176 |
| Condition 1B | 205 | 201 | 196 |
| Condition 2A | 186 | 185 | 184 |
| Condition 2B | 218 | 217 | 216 |
| Condition 3A | 172 | 195 | 208 |
| Condition 3B | 172 | 221 | 213 |
| Condition 4A | 208 | 234 | 233 |
| Condition 4B | 211 | 229 | 210 |

The A and B following the condition number represent two different pigs, each tested under four different ventilatory conditions (Conditions 1-4: two with healthy lungs and two with injured lungs). PaO₂ values remained adequate throughout all experimental conditions.

Table S4. pH values

| Experimental run | Pre-suctioning | During-suctioning | Post-suctioning |
| --- | --- | --- | --- |
| Condition 1A | 7.430 | 7.424 | 7.398 |
| Condition 1B | 7.434 | 7.440 | 7.425 |
| Condition 2A | 7.486 | 7.509 | 7.505 |
| Condition 2B | 7.500 | 7.515 | 7.542 |
| Condition 3A | 7.329 | 7.353 | 7.332 |
| Condition 3B | 7.369 | 7.403 | 7.405 |
| Condition 4A | 7.283 | 7.341 | 7.301 |
| Condition 4B | 7.343 | 7.372 | 7.320 |

The A and B following the condition number represent two different pigs, each tested under four different ventilatory conditions (Conditions 1-4: two with healthy lungs and two with injured lungs).

Figure S1. Schematic Diagram for Preliminary Experiments


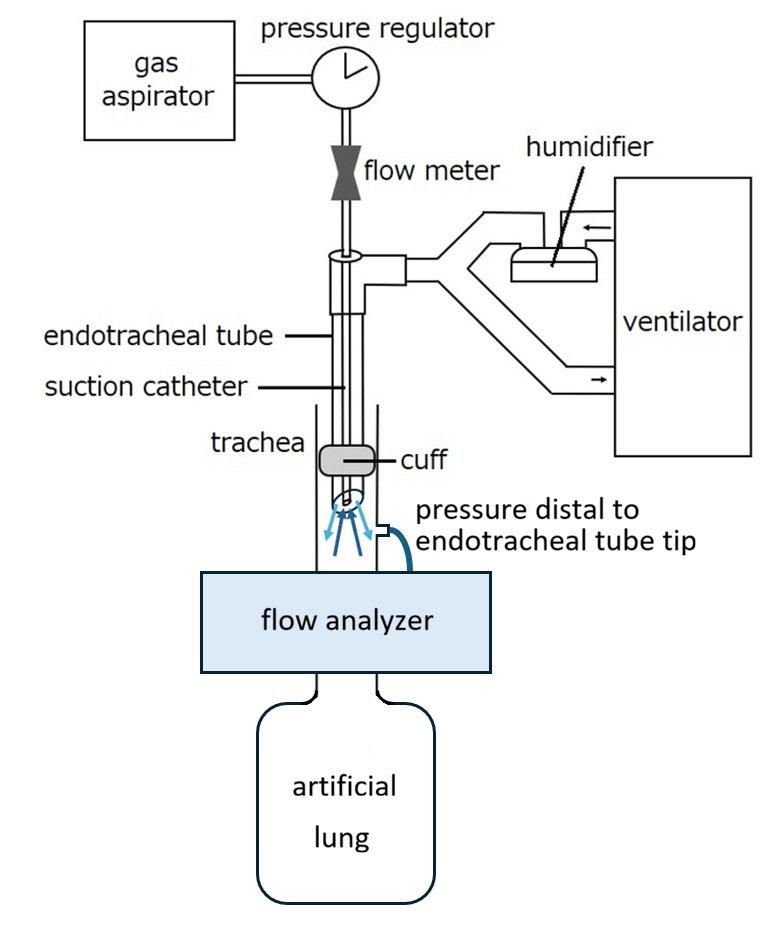


Figure S2. Preliminary experimental results: tidal volume measurements before and during continuous intratracheal suctioning


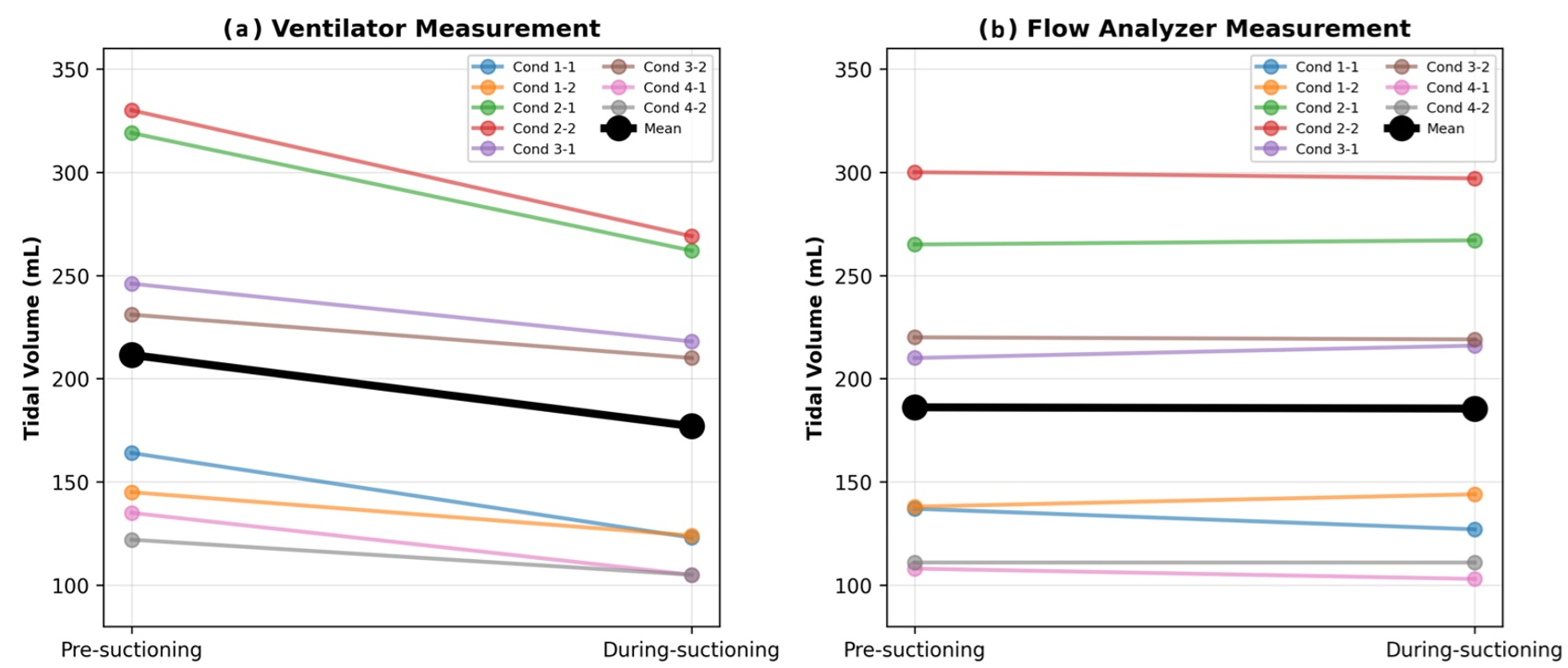


**(a) Ventilator-measured expiratory tidal volume** decreased significantly from 211.5 ± 82.6 mL to 177.0 ± 70.3 mL during continuous suctioning at 13-14 L/min, reflecting measurement artifact due to continuous intratracheal suctioning. **(b) Flow analyzer-measured actual expiratory tidal volume** remained unchanged (186.1 ± 73.1 mL vs. 185.5 ± 74.3 mL), confirming that THRICS operation effectively maintains actual tidal volume delivery. Individual measurements (colored lines) and mean values (black lines) for eight different experimental conditions (Condition 1-1 through Condition 4-2) are shown. Data are expressed as the mean ± SD.
